# Usefulness of cardio-magnetic resonance in the diagnosis and evaluation of pulmonary hypertension

**DOI:** 10.1186/1532-429X-13-S1-P335

**Published:** 2011-02-02

**Authors:** Pilar Egea-Serrano, Jeremy Collinst, Maria M Izquierdo, Timothy Scanlon, Philip Hodnett, Eduardo Segoviat, Manuel Sancho, James Carr

**Affiliations:** 1 Cardiology Department. Hospital de Cabueñes, Gijón, Spain; 2 Cardiovascular Imaging. Radiology Department. Northwestern Memorial Hospital., Chicago, IL, USA; 3 Cardiology Department. Northwestern Memorial Hospital., Chicago, IL, USA; 4 (1) Cardiovascular Imaging. Radiology Department. Northwestern Memorial Hospital, Chicago, IL, USA; 5 Cardiology Department. Hosital U Puerta del Mar, Cádiz, Spain

## Introduction

The diagnosis of PH should be based on clinical suspicious and specific complementary tests. CMR has no acoustic window limitation, it is the gold standard for quantification of right ventricular (RV) function, it is the main test for congenital heart disease (CHD) and finally it gives more comprehensive information.

## Purpose

**T**o show how useful the CMR was for patients referred for heart failure (HF), regarding the identification and severity of pulmonary hypertension (PH) not previously assessed by other non-invasive techniques.

## Methods

We reviewed patients referred to our institution from January 2008 to March 2010 for further evaluation of HF who were firstly diagnosed of PH by CMR. The study was performed on a 1.5T Avanto scanner and included: cine images, phase-contrast sequence, time-resolved angiography for aorta and pulmonary artery, and inversion recovery sequence. The data analyzed was: ventricular volumes and functions, flows, interventricular septum (IVS) motion, and assessment of late gadolinium enhancement (LGE).

## Results

20 patients were included. Eleven (55%) were female and the mean age was 47. Following the Dana Point classification 2008: 10 belonged to group 1; 4 to group 2; 1 to group 3; 1 to group 4; and 4 patients had mixed conditions. All patients presented high RV volumes (end-dyastolic 209 ± 88 mL; and end-systolic 128± 81,7 mL). The RV ejection fraction (RVEF) was 41 ± 16 %. The RV outflow tract was enlarged (30 ± 5 mm) and pulmonary artery (PA) too (36 ± 7,5 mm). RV wall thickness was 4,2± 1,4 mm. Pulmonary peak velocity was 105,7 ± 76,8 mmHg, with moderate regurgitation. IVS motion classified as: 50% flattening, 35 % D-shaped, normal only in 1. The left ventricular (LV) overall function was related to the baseline disease. The systolic RV/LV volume ratio was 1,9 ± 0,8 and the diastolic one was 1,6 ± 0,6. LGE was present in 60%, mostly in basal RV and LV insertion points; additionally in LV inferior wall in one patient suggesting myocardial infarct, and another patient presented transmural LGE in inferior apical wall. CMR angiography was of value as it allowed orthogonal measures of PA and showed features of chronic thromboembolic disease, pulmonary vein stenosis and discovered CHD.

## Conclusions

CMR is able not only to diagnose PH but also provide its severity and etiology, offering prognosis and guiding management / follow-up within a single examination.

